# Performance of Imaging Techniques in Non-invasive Diagnosis of Non-alcoholic Fatty Liver Disease in Children: A Systematic Review and Meta-Analysis

**DOI:** 10.3389/fped.2022.837116

**Published:** 2022-07-11

**Authors:** Qun Yu, Yiwei Liu, Peipei Hu, Feng Gao, Guoqing Huang

**Affiliations:** ^1^Department of Ultrasound, Affiliated Hospital of Hangzhou Normal University, Hangzhou, China; ^2^Hangzhou Normal University, Hangzhou, China

**Keywords:** elastography, imaging techniques, non-invasive diagnosis, non-alcoholic fatty liver disease, children

## Abstract

**Background and Aim:**

Non-alcoholic fatty liver disease (NAFLD) has become the most common chronic liver disease in children. With the continuous emergence of various non-invasive diagnostic methods, imaging techniques have always been considered as potential alternative methods to liver biopsy. This study aimed to evaluate the diagnostic performance of imaging techniques so as to search for the most promising technology.

**Methods:**

We searched English and Chinese databases. English databases included Cochran library, Embase, PubMed, and Web of Science, while Chinese databases included the Wanfang database and China National Knowledge Internet.

**Results:**

Finally, 11 articles were included (12 studies, one of which included studies on both fibrosis and steatosis). Further, 26.2% of the participants had mild steatosis, 34.1% had moderate steatosis, and 34.9% had severe steatosis. Also, 64.0% had any fibrosis, 29.1% had significant fibrosis, 13.8% had advanced fibrosis, and 2.8% had cirrhosis. Irrespective of the grade of fibrosis, transient elastography (TE) had higher sensitivity (97–100%), whereas magnetic resonance elastography (MRE) had the lowest sensitivity (58–63%). The pooled sensitivity and specificity of imaging techniques in diagnosing steatosis were 89% (95% CI, 71–96) and 89% (95% CI, 72–96), and AUROC 0.95 (95% CI, 93–97), multifrequency magnetic resonance elastography-hepatic fat fraction (mMRE-HFF) had the highest sensitivity (87%, 95% CI 77–97), ultrasonography (US) had the lowest specificity (96%, 95% CI 92–98%).

**Conclusion:**

Imaging techniques have a good diagnostic performance for children with NAFLD, especially the diagnosis of liver fibrosis based on ultrasound or magnetic resonance elastography. Compared with different imaging techniques, TE has the best performance in diagnosing significant fibrosis. Liver stiffness measurement (LSM) is expected to become a biological indicator for routine screening, dynamic monitoring of disease changes, and prognostic evaluation.

## Introduction

At present, obesity is a global epidemic, affecting approximately 20% of the global population (1). Among the widespread complications associated with obesity, the prevalence of non-alcoholic fatty liver disease (NAFLD) in children is 26.1 times that in normal weight children (1).

With the obesity epidemic (2, 3), the prevalence of NAFLD in children has more than doubled in the last 20 years (4), and it has also become the most common chronic childhood liver disease worldwide (5, 6). The prevalence of NAFLD in general children is 7.6%, while more than 34.2% in obese children (7). The latest 51-year long-term follow-up study showed that children with NAFLD had a higher all-cause mortality rate, which was 5.26 times that in the normal control group (8). Unfortunately, the natural history, pathogenesis, diagnosis, and treatment of pediatric NAFLD is not clear, considering the heavy medical and economic burden that it may bring and its impact on pediatric health. Therefore, we urgently need to pay great attention to this disease (5, 9–11).

The spectrum of NAFLD ranges from non-alcoholic fatty liver (NAFL) to non-alcoholic steatohepatitis (NASH), to liver fibrosis, to cirrhosis and liver cancer (12, 13). A previous study confirmed that 70% of pediatric NAFLD had fibrosis at the time of diagnosis (7). However, NAFLD usually has no obvious symptoms and remains silent (1). Pediatric NAFLD is a potentially progressive disease, which can progress to advanced liver fibrosis, cirrhosis, and liver-related transplantation diseases (8). A 20-year follow-up study showed that severe NAFLD recurred in the allogeneic body after liver transplantation, and again liver transplantation was still needed (8). Liver biopsy is the gold standard for diagnosing NAFLD. However, it is invasive, and the risk of fatal complications limits its application widely (14–16). Also, liver biopsy often bears higher risks and is less acceptable in children than in adults (17). Liver biopsy is obviously not suitable for those who need long-term observation of disease changes and treatment effects, especially for a large number of asymptomatic or mildly symptomatic children. Therefore, new non-invasive diagnostic methods are urgently required.

The commonly used diagnostic methods for NAFLD are alanine aminotransferase (ALT) and aspartate aminotransferase (AST), whereas the sensitivity and specificity of liver enzymes are relatively low (18, 19). Even under normal conditions of ALT, NAFLD can also progress to advanced fibrosis (15, 20). With the rapid development of imaging technology in the last 30 years, new technologies are introduced continually, especially elastography, which has been proven to quantitatively measure the hardness of liver tissue in the human body. Imaging techniques are the most promising non-invasive diagnostic methods. It has good diagnostic performance in adult NAFLD and has been used widely (21–26). However, few systematic studies explored the application of imaging techniques to diagnose NAFLD in children with liver biopsy as the gold standard.

The purpose of our study was to systematically evaluate and compare the diagnostic performance of imaging techniques based on hepatic histology through widely searching a large number of articles, and to find the most promising imaging diagnostic technology in pediatric NAFLD.

## Methods and Objectives

This systematic review and meta-analysis was based on the Preferred Reporting Items for Systematic Reviews and Meta-Analyses (PRISMA) diagnostic test criteria (27). It aimed to evaluate the diagnostic performance of new non-invasive imaging techniques in children with NAFLD so as to search for the most promising imaging technology.

### Search Strategy

The search ended on October 10, 2021. The English and Chinese databases were searched. The English database included Cochrane library, Embase, PubMed, and Web of Science, while the Chinese databases included the Wanfang database and China National Knowledge Internet. The medical subject heading terms used for the search included NAFLD, children, magnetic resonance, ultrasound, and liver transferase; and pediatric NAFLD and liver transferase, pediatric NAFLD and magnetic resonance; and pediatric NAFLD and ultrasound, finally connecting three retrieval formulas.

### Eligibility Criteria and Exclusion Criteria

The two researchers conducted independent searches after determining the complete search formula. First, they screened by title and abstract to exclude obviously irrelevant articles. For potentially qualified articles, we read through the full text, excluded articles that did not meet the standards, and finally articles that met the criteria were included. The final criteria were liver histopathology as the gold standard and diagnostic studies of ultrasound or magnetic resonance technology. During the search, if a dispute occurred between the two researchers, they negotiated with the third one to resolve it.

The exclusion criteria are as follows: non-children; non-human; non-diagnostic studies; no liver histopathology as the gold standard; non-English; non-Chinese; reviews; conferences; abstracts; guidelines; Chinese non-science citation index (SCI); incomplete data; or inability to construct a 2 × 2 diagnostic performance table.

### Data Extraction and Quality Assessment

The following data were extracted: author, publication year, region, research background, research time, research type, blinding method, characteristics of the included population (age, male- to -female ratio), characteristics of the included research test (test type, probe or machine model, test stage threshold, test success rate), histological scoring standards, histological grading, and correlation between test results and histology.

The quality of studies was assessed according to the Quality Assessment of Diagnostic Accuracy Studies-2 (QUADAS-2) standard (28).

### Staging System

The extent of fibrosis was uniformly divided into stages based on the NAFLD fibrosis histological score: no fibrosis (stage F0), any fibrosis (stage ≥ F1), significant fibrosis (stage ≥ F2), advanced fibrosis (stage ≥ F3), and cirrhosis (stage F4).

The extent of steatosis was uniformly divided into stages based on the NAFLD steatosis histological score: no steatosis (stage S0), mild steatosis (stage S1), moderate steatosis (stage S2), and severe steatosis (stage S3).

### Data Analysis

The quality of studies was evaluated according to the QUADAS-2 standard, and the chart was drawn. The bivariate mixed model was used to evaluate the sensitivity and specificity of the imaging technology in diagnosing pediatric NAFLD. The hierarchical summary receiver operating characteristic curve (HSROC) was used to draw the total ROC curve, and the 95% prediction threshold and 95% confidence interval were obtained. Cochran Q test and the inconsistency index (*I*^2^) were used to evaluate the level of heterogeneity between studies. *I*^2^ value > 50% indicated significant heterogeneity, and *I*^2^ value < 50% indicated slight heterogeneity; the smaller the *I*^2^ value, the smaller the heterogeneity. Deeks’ funnel plot and Deeks’ asymmetry test were used to evaluate publication bias; no publication bias was observed when *P* < 0.05.

Stata software version 12 and Review Manager software version 5.4 were used to analyze the data.

## Results

### Study Selection

**FIGURE 1:**
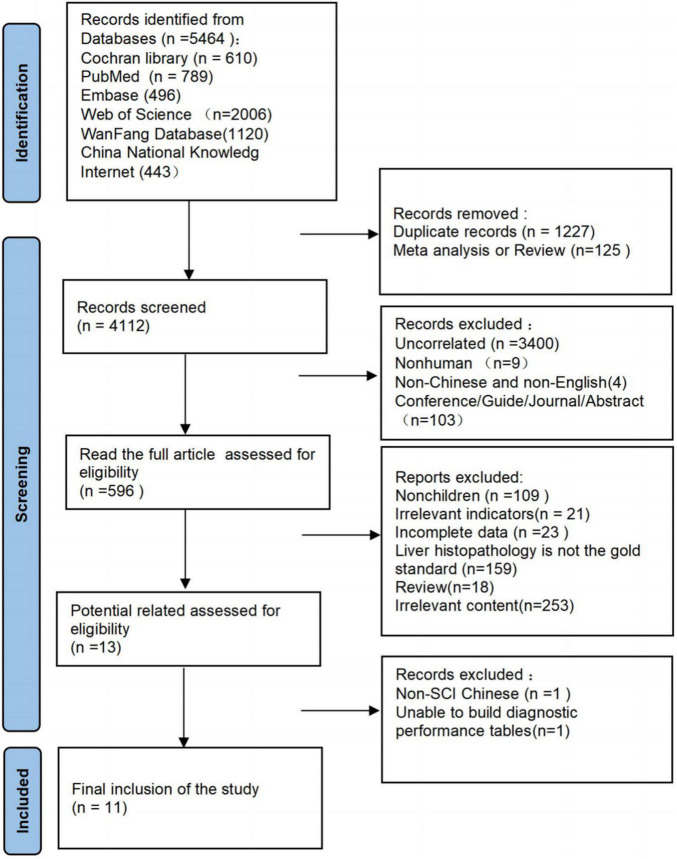
Flow diagram of study selection process.

As of October 10, 2021, 5464 documents were articles, 1227 duplicate articles and 125 reviews were excluded, and 3516 articles were excluded after screening titles and abstracts. Of these 3516 articles, 3400 were irrelevant (mainly focuses on the diagnosis or treatment of viral hepatitis, Wilson’s disease, and other liver diseases), 9 were non-human, 4 were non-Chinese and non-English, and 103 were conferences, guides, journals, or abstracts. The full text was read again to exclude 583 articles, including 109 articles for non-children, 21 other test indicators, 18 reviews, 23 articles with incomplete data, 253 irrelevant content,159 articles without liver histopathology as the standard, and further analysis of 13 articles for potentially qualified data. One article could not be used to construct a 2 × 2 diagnostic performance table, and one Chinese document was non-SCI (Figure 1).

### Study Characteristics

**FIGURE 2:**
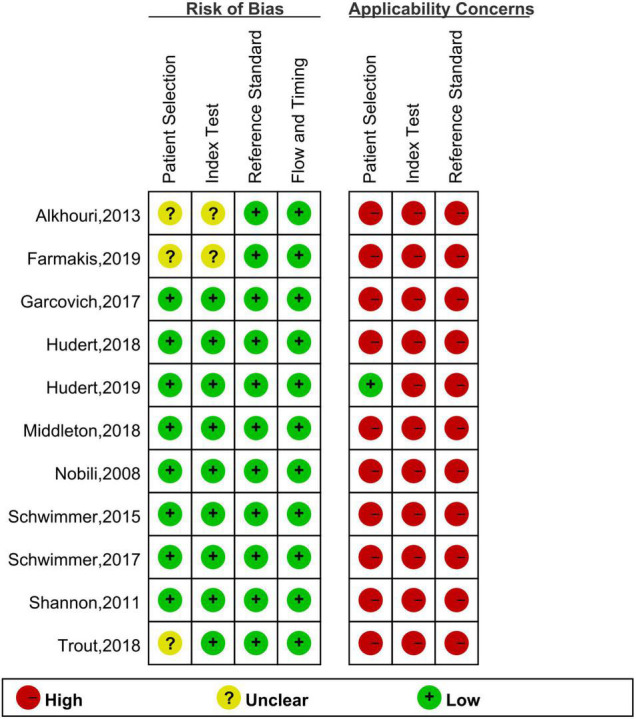
Quality assessment per study.

**FIGURE 3:**
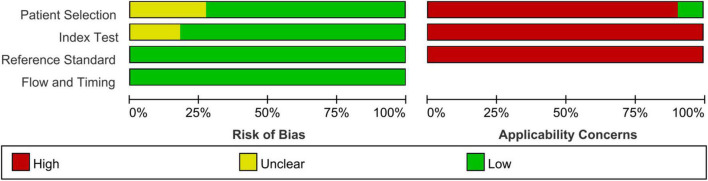
Summary of quality assessment.

**FIGURE 4:**
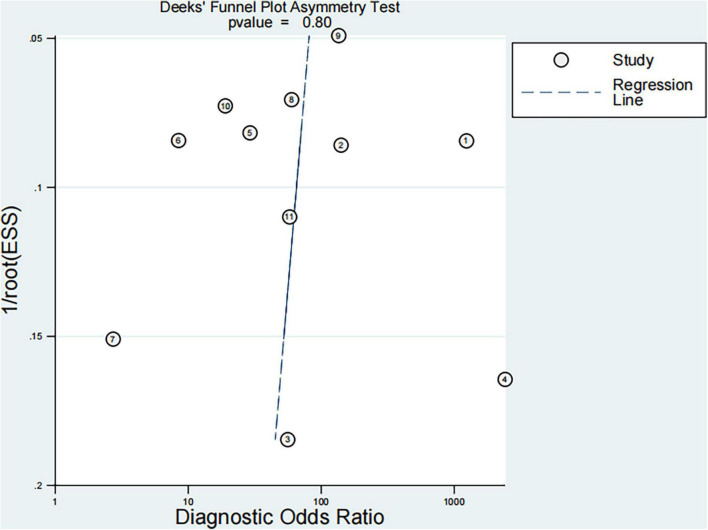
Publication bias.

Finally, 11 articles were included [12 studies, one article (29) included studies on both fibrosis and steatosis], comprising 8 prospective studies, 1 retrospective study, 9 studies using the blind method, 8 studies on fibrosis (29–36), 4 studies on steatosis (29, 37–39), 4 studies on magnetic resonance elastography (MRE) (1 MRE included both fibrosis and steatosis studies), 2 studies on magnetic resonance imaging-proton density fat fraction (MRI-PDFF), 2 studies on transient elastography (TE), 2 studies on shear wave elastography (SWE), 1 study on ultrasonography-time harmonic elastography (US-THE), and 1 study on conventional ultrasonography (US). Further, 11 studies staged the degree of fibrosis or steatosis, 11 studies had stage thresholds for imaging technology, and 7 studies had histology and imaging technology correlations. The success rate of 8 studies was between 84 and 100%, and the time interval between imaging technology and liver biopsy in 7 studies was 1–6 months. A total of 1011 children were enrolled in 12 studies in which all patients underwent liver biopsy. These children were mainly from tertiary hospitals, aged between 8 and 17 years (research characteristics are shown in Supplementary Tables 1, 2). Finally, the included articles had strong relevance, low bias (Figures 2, 3), and no publication bias (*P* = 0.80) (Figure 4).

### Prevalence of Fibrosis and Steatosis in Children With Non-alcoholic Fatty Liver Disease

In four studies on steatosis, 4.7% of the participants had no steatosis, 26.2% had mild steatosis, 34.1% had moderate steatosis, and 34.9% had severe steatosis. 36.3% of the five studies on liver fibrosis revealed no fibrosis (29, 30, 33, 34, 36), 64.0% of the six studies had any fibrosis (29, 30, 33–36), 29.1% of the seven studies had significant fibrosis (29–34, 36), 13.8% of five studies had advanced fibrosis (29, 30, 33, 34, 36), and 2.8% of two studies had cirrhosis (30, 34).

### Performance of Each Technique in Any Fibrosis (≥F1)

**FIGURE 5:**
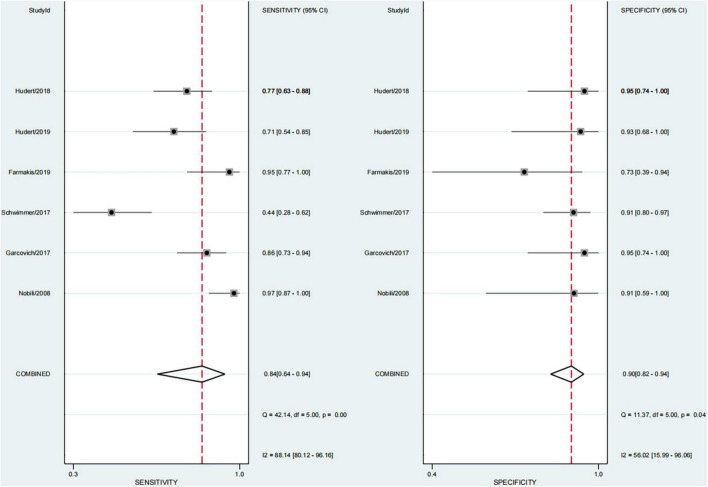
Sensitivity and specificity of elastography in the diagnosis of any fibrosis.

**FIGURE 6:**
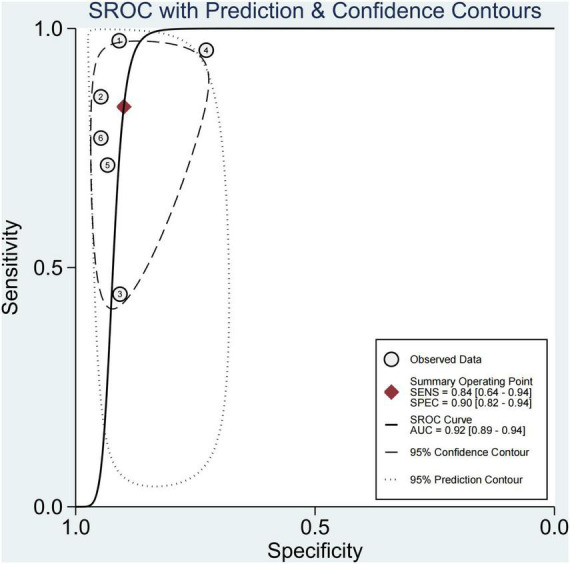
Summary ROC curve of diagnostic performance of elastography for any fibrosis.

In six studies, 64.0% of the participants had fibrosis (29, 30, 33–36). The pooled sensitivity and specificity of elastography were 84% (95% CI, 64–94) and 90% (95% CI, 82–94), respectively, and AUROC 0.92 (95% CI, 89–94) (Figures 5, 6). The sensitivity of TE was the highest at 97% (95% CI, 87–100), and the sensitivity of MRE was the lowest at 58% (95% CI, 45–69). Ultrasonography-time harmonic elastography (US-THE) had the highest specificity of 95% (95% CI, 74–100) (Supplementary Table 3).

### Performance of Each Technique in Significant Fibrosis (≥F2)

**FIGURE 7:**
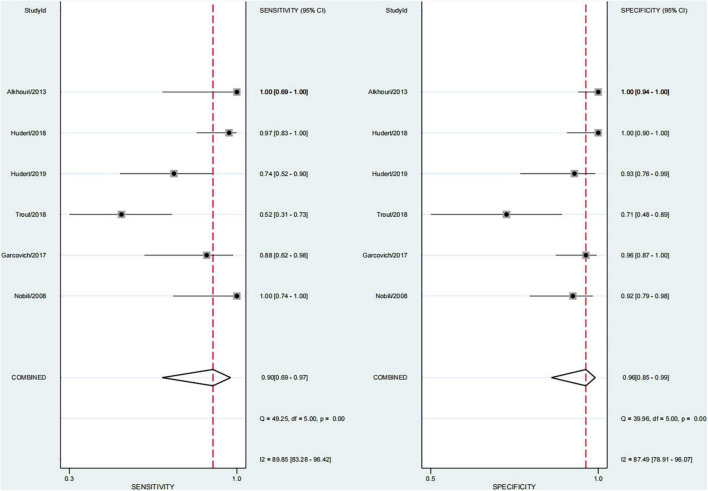
Sensitivity and specificity of elastography in the diagnosis of significant fibrosis.

**FIGURE 8:**
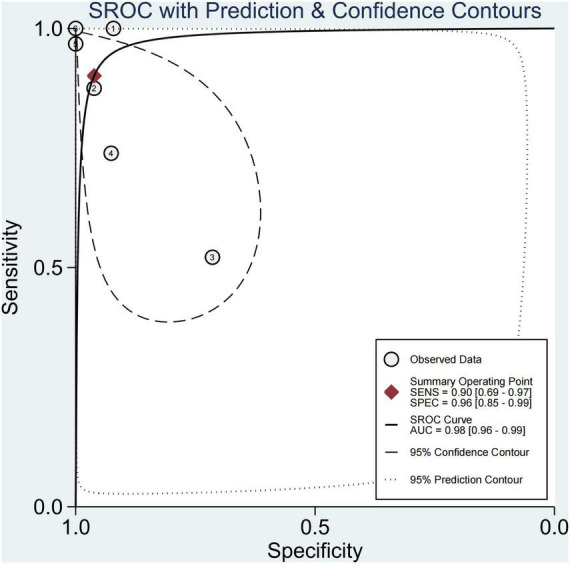
Summary ROC curve of diagnostic performance of elastography for significant fibrosis.

Finally, this study found that 29.1% of children had significant fibrosis in six studies (29–33, 36). The pooled sensitivity and specificity for diagnosing of significant fibrosis were 90% (95% CI, 69–97) and 96% (95% CI, 85–99), and AUROC 0.98 (95% CI, 96–99) (Figures 7, 8). The sensitivity of TE was the highest at 100% (95% CI, 74–100). The specificity of US-THE was the highest at 100% (95% CI, 90–100). The sensitivity and specificity of MRE were the lowest, at 63% (95% CI, 48–77) and 83% (95% CI, 70–93), respectively (Supplementary Table 4).

### Performance of Each Technique in Advanced Fibrosis (≥F3)

**FIGURE 9:**
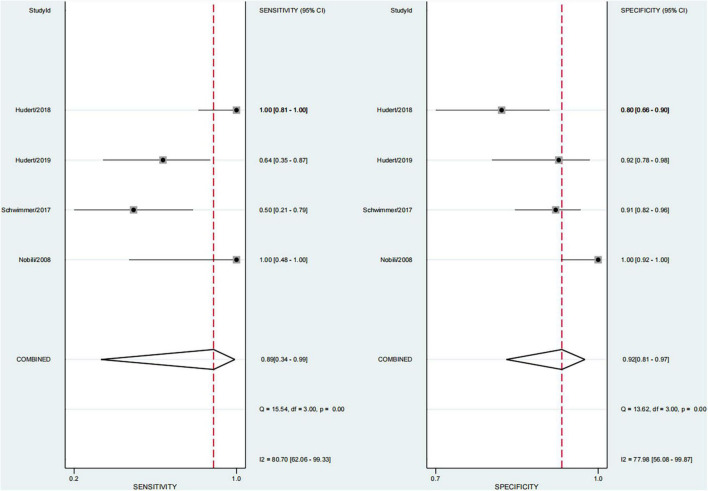
Sensitivity and specificity of elastography in the diagnosis of advanced fibrosis.

**FIGURE 10:**
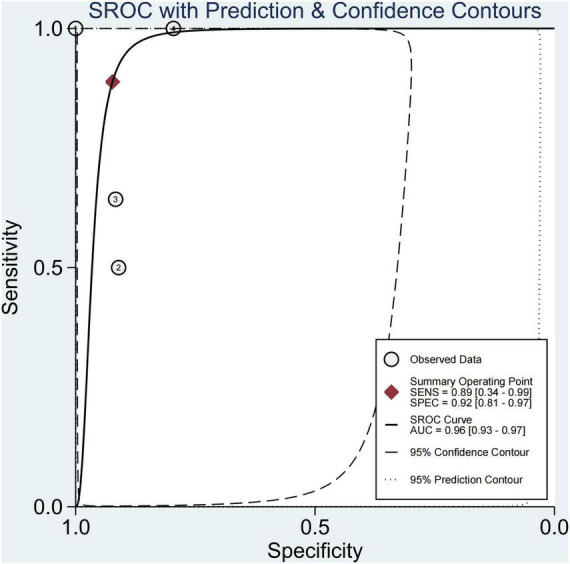
Summary ROC curve of diagnostic performance of elastography for advanced fibrosis.

This study found that 13.8% of children had advanced fibrosis in four studies (29, 30, 34, 36). The pooled sensitivity and specificity for diagnosing of advanced fibrosis were 89% (95% CI, 34–99) and 92% (95% CI, 81–97), and AUROC 0.96 (95% CI, 93–97) (Figures 9, 10). TE and US-THE had the highest sensitivity at100% (95% CI, 48–100) and 100% (95% CI, 81–100), respectively. TE had the highest specificity at 100% (95% CI, 92–100), while MRE had the lowest sensitivity at 58% (95% CI, 37–77) (Supplementary Table 5).

### Performance of Each Technique in Cirrhosis (F4)

The study found that four children (2.8%) had cirrhosis in two studies (30, 34). Hence, we could not calculate sensitivity, specificity, and AUROC.

### Performance of Each Technique for Diagnosing Steatosis

**FIGURE 11:**
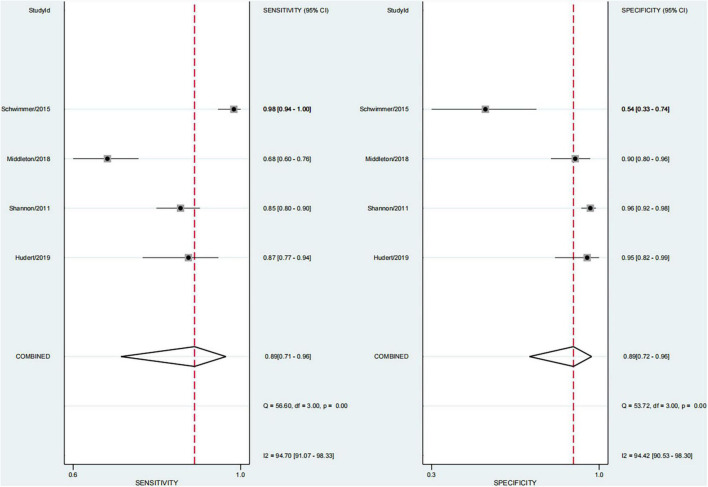
Sensitivity and specificity of imaging techniques for the diagnosis of steatosis.

**FIGURE 12:**
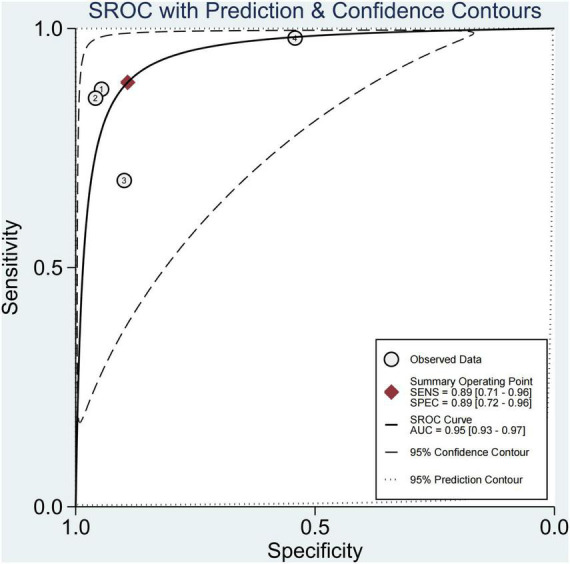
Summary ROC curve of diagnostic performance of image techniques for steatosis.

In four studies on steatosis (29, 37–39), 4.7% of the participants had no steatosis, 26.2% had mild steatosis, 34.1% had moderate steatosis, and 34.9% had severe steatosis. The pooled sensitivity and specificity for the diagnosis of steatosis were 89% (95% CI, 71–96) and 89% (95% CI, 72–96), and AUROC 0.95 (95% CI, 93–97) (Figures 11, 12). The sensitivity of multifrequency MRE-hepatic fat fraction (mMRE-HFF) was slightly better than that of MRI-PDFF and US, while the specificity of US was slightly higher than that of mMRE-HFF and MRI-PDFF (Supplementary Table 6).

### Heterogeneity Analysis

This study found that the heterogeneity (*I*^2^) in diagnosing NAFLD in children was significant (92.8 and 82.5%, respectively). Therefore, we conducted a subgroup analysis and found that the heterogeneity showed a downward trend (when diagnosing advanced fibrosis, *I*^2^ of sensitivity and specificity was 80.7 and 77.98%, respectively). Therefore, we analyzed the cause of high heterogeneity. A few studies used liver histology as the gold standard in pediatric NAFLD. As a result, a variety of imaging techniques were included in the study, the publication time span was large, and the sample size of each study varied greatly. We could not calculate the stage threshold of each technology precisely because of the small number of studies, the variety of imaging technologies involved, and the existence of heterogeneity. However, whether it was quantitative or qualitative, we study found that the imaging technologies had the diagnostic potential to replace the gold standard of liver biopsy in diagnosing children with NAFLD.

## Discussion

We searched a large number of articles. A total of 5464 articles were retrieved, and 11 articles were finally included, with a total impact factor of 120.203. According to the QUADAS-2 standard, through evaluating quality and drawing a diagram, we found high quality, low bias, and no publication bias (*P* = 0.80).

The study found that the imaging techniques had a good diagnostic performance for children with NAFLD, especially elastography technology. Whether it was based on ultrasound or magnetic resonance elastography, liver stiffness measurement (LSM) based on different elastography techniques was a good biological indicator, which was consistent with previous findings (40–42).

However, LSM is affected by various factors such as obesity, various liver diseases, elevated bilirubin, or diet. Therefore, comprehensive evaluation and confounding factors should be considered in diagnosing pediatric NAFLD with fibrosis. Currently, ALT was considered to be the best biomarker for screening NAFLD due to its low price, minimal invasiveness, and acceptable sensitivity (12, 43), Mark et al. used liver MRI-PDFF ≥ 18% as the standard for diagnosing severe fatty liver, and found that the serum ALT level was abnormal in 12 of 13 cases (44). No uniform threshold was available for diagnosing NAFLD in children due to restrictions such as sex, ethnicity, and dietary habits (5), and controversy existed in the limited number of studies. He et al. found that the serum upregulated levels of cytokeratin-18 (CK-18) and fibroblast growth factor-21 (FGF-21) correlated with the increased risk of NASH in children (45). However, the diagnostic accuracy of the two serum indexes was unsatisfactory, with currently no valid cutoffs. Magnetic resonance-based elastography techniques were time consuming, expensive, and difficult for children to cooperate (18). Moreover, ultrasound-based elastography is easy to operate, is more friendly to children, and has good compliance (5). Especially two-dimensional SWE (2D-SWE) can help observe the changes in liver parenchyma in real time (42) and effectively avoid the influence of liver blood vessels and tumors when measuring LSM (46). Compared with the low sensitivity and specificity of ALT and the underdiagnosis of children with NAFLD having a normal ALT level (47), LSM is expected to be better in routine screening and dynamic monitoring of disease changes and as a biological indicator for evaluating prognosis (40, 46). However, LSM is mainly associated with liver fibrosis and has a limited diagnostic value for children with NASH without liver fibrosis.

Pediatric NAFLD is a progressive disease. It may progress to liver fibrosis, and even cirrhosis and related hepatocellular carcinoma (13, 48, 49). Hence, the existence of fibrosis may be an important prognostic factor for disease progression and complications (43). In our study, the findings of 64.0% of children combined with fibrosis were consistent with previous studies (11, 40). How to accurately diagnose the presence of fibrosis and staging of fibrosis is a hot topic in the diagnosis and research of children with NAFLD. Liver biopsy is the gold standard, but it has many disadvantages; it is invasive and not suitable for repeated use, limiting the evaluation of disease progress in children with NAFLD. This study found that the sensitivity and specificity of elastography to diagnose any fibrosis were 84% (95% CI, 64–94) and 90% (95% CI, 82–94), respectively, and AUROC 0.92 (95CI, 89–94), with good diagnostic performance. We further found the sensitivity of TE was 97% (95% CI, 87–100). However, the application of TE for extremely obese children was limited (42, 50). The US-THE technology could be used to generate shear waves in the liver through external excitation to obtain the LSM of the entire liver, and was not limited by obesity (36).

At present, simple NAFL is considered harmless, and the high risk is related to NASH and advanced liver fibrosis (51). This study found that 29.1% of pediatric NAFLD had significant fibrosis, close to 30%, which was slightly lower than the 40% significant fibrosis reported by Xiao et al. (52), which was worthy of our vigilance. Therefore, new technologies and methods need to be developed to screen these high-risk children. In the last 30 years, imaging technology has developed rapidly. However, systematic reviews on the application of imaging technology to evaluate NAFLD in children with significant fibrosis are lacking. This meta-analysis found that the sensitivity and specificity for diagnosing significant fibrosis were 90% (95% CI, 69–97) and 96% (95% CI, 85–99). Among them, TE had the highest sensitivity at 100% (95% CI, 74–100), and MRE had the lowest sensitivity and specificity: 63% (95% CI, 48–7%) and 83% (95% CI, 70–93), respectively. Studies have found that ultrasound-based elastography and magnetic resonance-based elastography is effective in diagnosing significant fibrosis in children with NAFLD, with an AUROC of 0.98 (95% CI, 96–99). TE has the highest sensitivity and specificity. Our findings were consistent with the results of Draijer et al. (53). However, many studies in the last 10 years have confirmed that 2D-SWE is no less effective in diagnosing liver fibrosis compared with TE, and has the advantages of high detection success rate, large sampling range, and 2D visualization sampling, thus having good clinical application prospects (54, 55). Mǎrginean et al. also believed that the two methods of TE and 2D-SWE could be used for the non-invasive assessment of obesity-related liver fibrosis in children (56). Moreover, for patients who could not be diagnosed in the gray area of imaging examination, the serum biomarkers had a certain supplementary diagnostic value. Perhaps the combined application of imaging techniques and serum biomarkers is a better way to diagnose NAFLD in children, which is worthy of further research.

This study found that advanced fibrosis accounted for 13.8%, which was similar to that reported in the literature (40), but slightly lower than the 15–20% advanced fibrosis reported by Goldner et al. (11). This study found that the sensitivity and specificity in diagnosing advanced fibrosis were 89% (95% CI, 34–99) and 92% (95% CI, 81–97), and AUROC 0.96 (95% CI, 93–97). Among them, TE and US-THE had the highest sensitivity at 100% (95% CI, 48–100) and 100% (95% CI, 81–100), respectively. The specificity of TE was the highest at 100% (95% CI, 92–100), followed by MRE at 91% (95% CI, 84–96). In addition, other studies have also found that the application of TE can detect the severity of liver fibrosis (57). However, in a meta-analysis of adult NAFLD, the sensitivity and specificity of TE in diagnosing advanced liver fibrosis were 87 and 79%, respectively, and the AUROC was 88%, which was lower than that for the diagnosis in children. The sensitivity and specificity of SWE and MRE in diagnosing advanced fibrosis were 90 and 93%, and 84 and 90%, respectively, and the summary AUROC was 95 and 96%, respectively, which were higher than the diagnostic performance in children with NAFLD (52). Moreover, TE diagnosis of pediatric NAFLD with advanced fibrosis was reported only in a single-center study, requiring further verification. Some studies suggested that the diagnostic performance of acoustic radiation force impulse (ARFI) in patients with advanced liver fibrosis was similar to that of TE and SWE (58), but the failure rate of the technique increased with obesity and the ARFI value was affected by inflammation and necrotic tissue. Hence, the reliability of the results remains to be confirmed.

Without intervention in the fibrosis stage of NAFLD, cirrhosis also could occur in childhood. The prevalence of liver cirrhosis ranged from 0 to 10% (43). This was consistent with our finding that 2.8% of children had cirrhosis, but we could not find the best diagnostic technique due to the limitation of the number of studies.

We also found that the prevalence of severe steatosis was close to 35%, and only 4.7% of children had no steatosis. The pooled sensitivity and specificity of imaging techniques in diagnosing steatosis were 89% (95% CI, 71–96) and 89% (95% CI, 72–96). The sensitivity of mMRE-HFF was slightly better than that of MRI-PDFF and US, and the specificity of US was slightly higher than that of mMRE-HFF and MRI-PDFF. A few studies have shown that imaging techniques perform well in diagnosing steatosis, with an AUROC of 0.95 (95% CI, 93–97). mMRE-HFF had better sensitivity while US had higher specificity. Some studies had confirmed that PDFF was the leading non-invasive biomarker for liver fat quantification, and its diagnostic performance was superior to that of the controlled attenuation parameter (CAP) (59–61). However, the high cost of PDFF and mMRE-HFF limited their wide application. Meanwhile, the laboratory parameters also could not accurately predict the extent of liver fat infiltration.

We acknowledge that the study had some limitations. (1) The number of studies was small, which was related to the use of liver histology as the gold standard to ensure the quality of articles. The search found few studies using the non-invasive imaging diagnostic method of pediatric NAFLD with liver biopsy as the standard, hence highlighting the need for further follow-up research. (2) The population was mostly from tertiary hospitals, and the prevalence of fibrosis could not be extrapolated to the general children. Taking into account different ethnicities and races, the natural history of pediatric NAFLD could not be analyzed. Therefore, epidemiological investigations of long-term effects are still needed. (3) Researches conducted on each imaging technology are few, and hence large-sample multi-center research is needed for verification in the future. (4) High heterogeneity. We could not calculate the stage threshold of each technology precisely, because a variety of imaging techniques were included in the study, the publication time span was large, and the sample size of each study varied greatly. Different evaluation methods with different technicalities, differences in the equipment manufacturing companies, and so forth further affected our determination of the cutoff value. However, it still cannot be denied that imaging technology is a promising non-invasive diagnostic tool for children with NAFLD.

In summary, imaging techniques showed a good diagnostic performance for children with NAFLD, especially the diagnosis of fibrosis with the use of ultrasound-based elastography and magnetic resonance-based elastography. Compared with different imaging techniques, TE had the best performance in diagnosing significant fibrosis in pediatric NAFLD. Ultrasound-based elastography technology was easy to operate and more friendly for children and had good compliance. Hence, it can be used as the most promising non-invasive imaging technology for the diagnosis and screening of high-risk children with NAFLD. LSM is expected to be a promising biological indicator for routine screening and dynamic monitoring of disease changes and prognostic evaluation.

### What Is Known

1. NAFLD is the most common chronic liver disease in children.
2. New and effective non-invasive diagnostic tools are urgently needed.
3. Elastography technology showed a good performance in diagnosing adult NAFLD.

### What Is New

1. Imaging techniques showed a good diagnostic performance for children with NAFLD, especially in diagnosing children with fibrosis by ultrasound-based elastography and magnetic resonance-based elastography. Compared with different imaging techniques, TE showed the most promising performance in diagnosing significant fibrosis in pediatric NAFLD, but further verification is needed.
2. Ultrasound-based elastography is easy to operate and has good compliance for children, which can be used as the preferred non-invasive imaging technology for the diagnosis and screening of high-risk children with NAFLD. LSM is expected to be a promising biological indicator for routine screening, dynamic monitoring of disease changes, and prognostic evaluation.

## Data Availability Statement

The raw data supporting the conclusions of this article will be made available by the authors, without undue reservation.

## Author Contributions

QY wrote the manuscript. YL performed the statistical analysis. PH performed the statistical analysis and analyzed the data. GH and FG provided ideas and analyzed the data. All authors contributed to the article and approved the submitted the final version.

## Conflict of Interest

The authors declare that the research was conducted in the absence of any commercial or financial relationships that could be construed as a potential conflict of interest.

## Publisher’s Note

All claims expressed in this article are solely those of the authors and do not necessarily represent those of their affiliated organizations, or those of the publisher, the editors and the reviewers. Any product that may be evaluated in this article, or claim that may be made by its manufacturer, is not guaranteed or endorsed by the publisher.

## Abbreviations

NAFLD: non-alcoholic fatty liver disease
NAFL: non-alcoholic fatty liver
NASH: non-alcoholic steatohepatitis
LSM: liver stiffness measurement
95% CI: 95% confidence interval
AUROC: area under the receiver operating characteristic curve
MRE: magnetic resonance elastography
TE: transient elastography
SWE: shear wave elastography
US-THE: ultrasonography-time harmonic elastography
mMRE-HFF: multifrequency magnetic resonance elastography-hepatic fat fraction
MRI-PDFF: magnetic resonance imaging-proton density fat fraction
US: ultrasonography
2D-SWE: two dimensional-shear wave elastography
ALT: alanine aminotransferase
AST: aspartate aminotransferase
CK18: cytokeratin 18
FGF-21: fibroblast growth factor-21
CAP: controlled attenuation parameter.
