# Supplementary figures and images for: Performance of Imaging Techniques in Non-invasive Diagnosis of Non-alcoholic Fatty Liver Disease in Children: A Systematic Review and Meta-Analysis
Source: Front Pediatr. 2022 Jul 11;10:837116. doi: 10.3389/fped.2022.837116 (PMC9311375; doi:10.3389/fped.2022.837116)

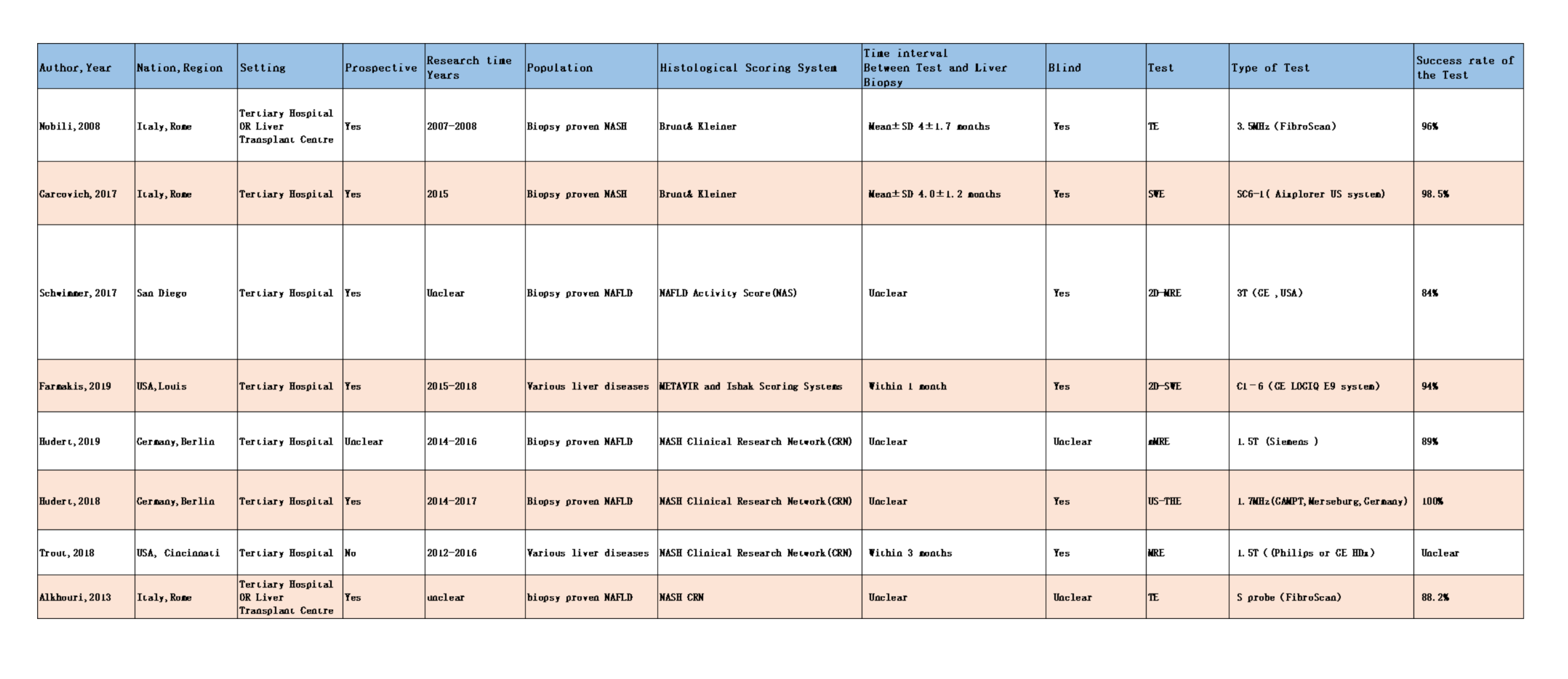

Supplement: Supplementary file 1 [file Data_Sheet_1.zip › Table/Table 1(A) Research characteristics of fibrosis.tif]

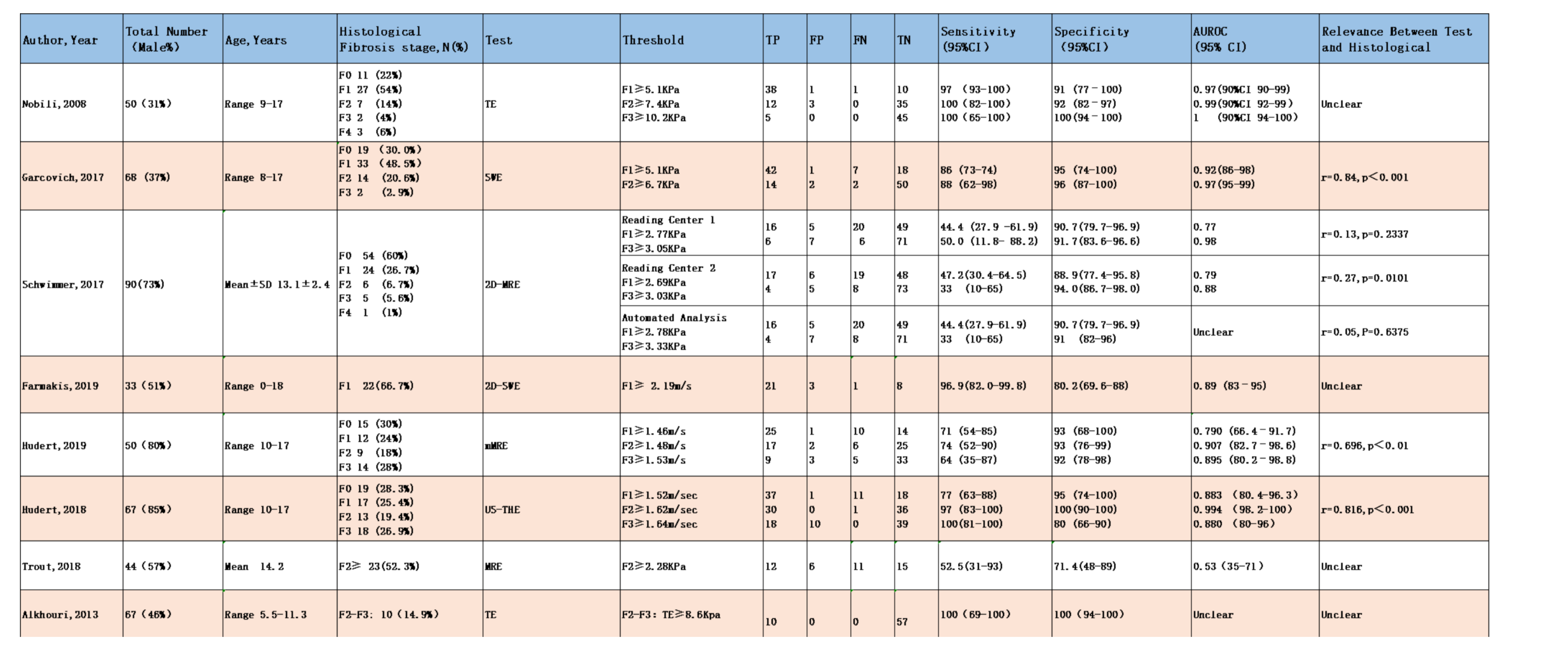

Supplement: Supplementary file 1 [file Data_Sheet_1.zip › Table/Table 1(B) Research characteristics of fibrosis.tif]

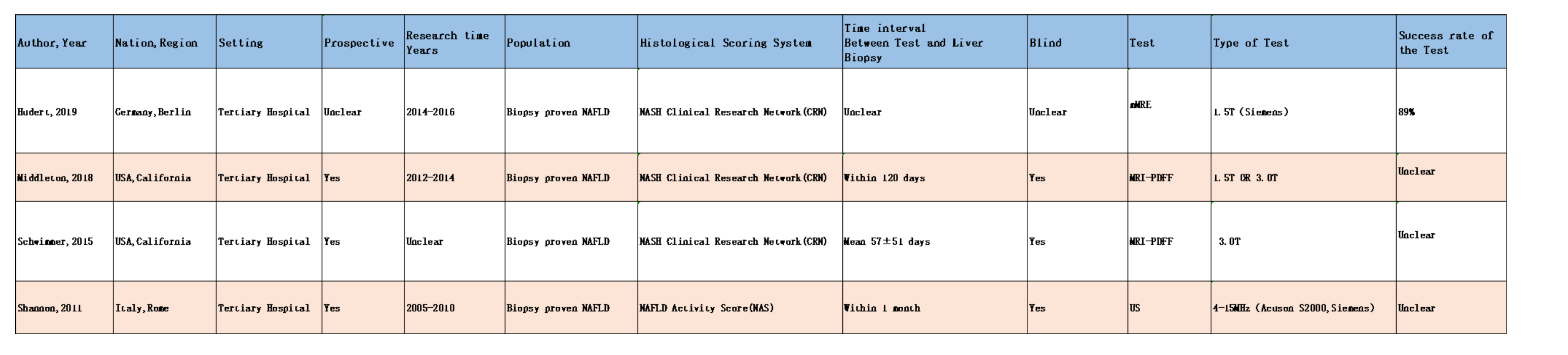

Supplement: Supplementary file 1 [file Data_Sheet_1.zip › Table/Table 2(A)Research characteristics of steatosis.tif]

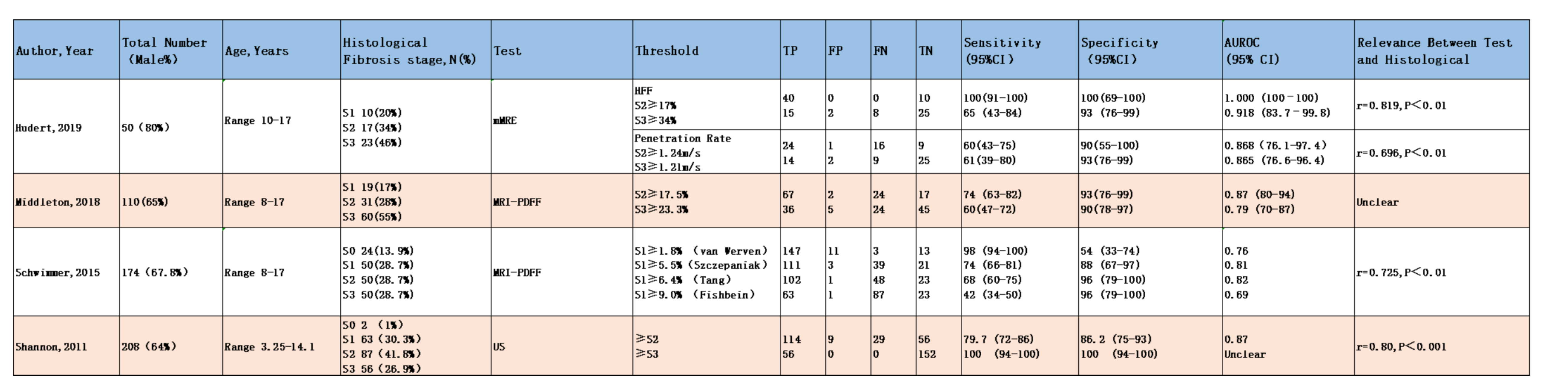

Supplement: Supplementary file 1 [file Data_Sheet_1.zip › Table/Table 2(B)Research characteristics of steatosis.tif]
