# Supplementary material for: Performance of Imaging Techniques in Non-invasive Diagnosis of Non-alcoholic Fatty Liver Disease in Children: A Systematic Review and Meta-Analysis
Source: Front Pediatr. 2022 Jul 11;10:837116. doi: 10.3389/fped.2022.837116 (PMC9311375; doi:10.3389/fped.2022.837116)
Supplement: Supplementary file 1 [file Data_Sheet_1.zip › Table/Table 3,4,5,6.doc]

**Table 3 Sensitivity and specificity of each elastography technique in the diagnosis of any fibrosis (≥F1)**

| I[mage](../../../../C:/Users/HUAWEI/AppData/Local/youdao/dict/Application/8.10.3.0/resultui/html/index.html#/javascript:;) [technology](../../../../C:/Users/HUAWEI/AppData/Local/youdao/dict/Application/8.10.3.0/resultui/html/index.html#/javascript:;) | Number | Sensitivity(95%CI) | Specificity(95%CI) |
| --- | --- | --- | --- |
| TE | 1 | 97% (87-100) | 91% (59-100) |
| MRE | 2 | 58% (45-69) | 91% (82-97) |
| SWE | 2 | 89% (79-95) | 87% (69-96) |
| US-THE | 1 | 77% (63-88) | 95% (74-100) |

Abbreviations: TE, transient elastography; MRE, magnetic resonance elastography; SWE, shear wave elastography; US-THE, ultrasonography- time-harmonic elastography

**Table 4 Sensitivity and specificity of each elastography technique in the diagnosis of significant fibrosis (≥F2)**

| I[mage](../../../../C:/Users/HUAWEI/AppData/Local/youdao/dict/Application/8.10.3.0/resultui/html/index.html#/javascript:;) [technology](../../../../C:/Users/HUAWEI/AppData/Local/youdao/dict/Application/8.10.3.0/resultui/html/index.html#/javascript:;) | Number | Sensitivity(95%CI) | Specificity(95%CI) |
| --- | --- | --- | --- |
| TE | 2 | 100%(74-100) | 97% (91-99) |
| MRE | 2 | 63% (48-77) | 83% (70-93) |
| SWE | 1 | 88% (62-98) | 96% (87-100) |
| US-THE | 1 | 97% (83-100) | 100%(90-100) |

Abbreviations: TE, transient elastography; MRE, magnetic resonance elastography; SWE, shear wave elastography; US-THE, ultrasonography- time-harmonic elastography

**Table 5 Sensitivity and specificity of each elastography technique in the diagnosis of advanced fibrosis (≥F3)**

| I[mage](../../../../C:/Users/HUAWEI/AppData/Local/youdao/dict/Application/8.10.3.0/resultui/html/index.html#/javascript:;) [technology](../../../../C:/Users/HUAWEI/AppData/Local/youdao/dict/Application/8.10.3.0/resultui/html/index.html#/javascript:;) | Number | Sensitivity(95%CI) | Specificity(95%CI) |
| --- | --- | --- | --- |
| TE | 1 | 100%(48-100) | 100%(92-100) |
| MRE | 2 | 58% (37-77) | 91% (84-96) |
| US-THE | 1 | 100%(81-100) | 80% (66-90) |

Abbreviations: TE, transient elastography; MRE, magnetic resonance elastography; US-THE, ultrasonography- time-harmonic elastography

**Table 6 The overall sensitivity and specificity of each image technique in the diagnosis of steatosis**

| I[mage](../../../../C:/Users/HUAWEI/AppData/Local/youdao/dict/Application/8.10.3.0/resultui/html/index.html#/javascript:;) [technology](../../../../C:/Users/HUAWEI/AppData/Local/youdao/dict/Application/8.10.3.0/resultui/html/index.html#/javascript:;) | Number | Sensitivity(95%CI) | Specificity(95%CI) |
| --- | --- | --- | --- |
| mMRE-HFF | 1 | 87% (77-97) | 95% (82-99) |
| MRI-PDFF | 2 | 83% (78-87) | 81% (71-88) |
| US | 1 | 85% (80-90) | 96% (92-98) |

Abbreviations: mMRE-HFF, multifrequency magnetic resonance elastography -hepatic fat fraction; MRI-PDFF, magnetic resonance imaging- proton density fat fraction;

US, ultrasonography
